# How Small is Too Small?

**DOI:** 10.21470/1678-9741-2025-0276

**Published:** 2025-11-17

**Authors:** Davi Freitas Tenorio, Lee Morgan Fuentes, Charles Duncan Fraser Jr.

**Affiliations:** 1 Department of Cardiovascular and Thoracic Surgery, Division of Pediatric and Congenital Cardiothoracic Surgery, University of Texas at Austin Dell Medical School, Austin, Texas, United States of America

## Central Message:

Cardiac surgery in micronatal neonates - under 2.5 kilograms - defines the cutting edge of congenital heart surgery. Success hinges on institutional readiness, relentless teamwork, and the humility to know when innovation must yield to pragmatism.

At a time when congenital heart surgery already demands extraordinary technical acumen and emotional endurance, the emergence of cardiac procedures in micronatal neonates presents an even more formidable frontier. These patients test not only the boundaries of physiology and surgical finesse but also the infrastructure and philosophy of institutions that dare to care for them. What was once rare and daunting is now increasingly confronted in Neonatal Intensive Care Units (NICUs) and surgical suites across the world. But the question remains: how small is too small?

In this editorial, we present insights from our team, whose experience in Austin sheds sobering and instructive light on the evolving field of micronatal cardiac surgery. The report outlines not just surgical strategies, but also the existential calculus institutions must perform when confronting cases that stretch the limits of current capabilities. While the data and reflections are from one center, the implications are global.

## The Defining Variable: Weight, Lesion, and Timing

In clinical practice, there are few more anxiety-inducing moments than when a prenatal diagnosis reveals a serious cardiac defect in a fetus estimated at < 2 kilograms. The usual algorithmic decisiveness gives way to case-by-case deliberation: wait or operate^[1]^, palliate or repair^[2]^, feed and grow, or intervene early^[3]^?

The lesion dictates urgency. Truncus arteriosus with severe insufficiency rarely waits. Interrupted aortic arch with ventricular septal defect might allow for temporization - though not always. As the authors describe, the so-called "feed and grow" strategy, once a convenient default, is being reassessed. What is the optimal target weight before surgery? Is it 1800 grams? 2000 grams? And what are the physiological costs of waiting?

It is this triad - weight, anatomy, and time - that defines the precarious balance. With prostaglandin exposure extending into weeks and nutritional goals often unmet, the window for a “safer” operation may never arrive. This is where institutional philosophy meets surgical realism.

## Institutional Capability Is Not Just Capacity

What distinguishes an institution capable of performing micronatal surgery is not its census of NICU beds or the square footage of its operating room. Rather, it is its system-wide coherence: a seamless choreography among neonatologists, anesthesiologists, perfusionists, nurses, and surgeons. Even transporting an 800-gram baby is a high-stakes endeavor. Hypothermia, desaturation, line displacement - each threatens the outcome before the first incision and must be anticipated and managed precisely.

In this setting, the anesthesia team becomes critical^[4]^. Machines must deliver micro-precision tidal volumes. Lines must be pre-primed with obsessive accuracy. There is no room for approximations or improvisation. An 800-gram neonate holds < 80 mL of blood - every drop, every bolus, every heparinized flush counts.

Cardiopulmonary bypass must also be adapted^[5]^. Smaller roller heads, reduced tubing lengths, and nitrogen management in gas exchange circuits are not mere modifications - they are prerequisites. Just as importantly, surgical success can be quickly undone by a misstep in the intensive care unit (ICU), whether due to ventilator error or miscommunication in handoff. Flawless continuity of care is as important as flawless technique.

## The Myth of One-and-Done: A Team Sport from End to End

In these challenging cases, it is clear that surgical brilliance is necessary but insufficient. These procedures require a culture of shared vigilance. From preoperative planning through recovery, the team must function as a single organism.

Postoperative insights from neonatology - often undervalued in cardiac centers - are fundamental. A seemingly minor adjustment, like prone positioning, turned the tide for one patient with persistent pulmonary compromise before repair. Such details, discovered and shared through inter-service trust, often make the difference between survival and attrition^[6]^.

## The Uncomfortable Truths: Limits and Leadership

From our experience, one of the most candid observations that can be made is this: for micronates, there is often only one chance to get it right. There is no meaningful margin for error - technical, systemic, or cognitive. As one seasoned surgeon commented, “a mistake in these cases is very hard to recover from”. That statement, stark as it is, must resonate with training programs, hospital leaders, and policymakers alike.

Not every center can or should perform these operations. Institutional ego must be tempered by surgical humility. While it is commendable to build a program capable of such feats, it is even more honorable to know when referral is the safer path. Conversely, for centers with the vision, patience, and infrastructure to pursue this mission, the responsibility is immense. It is not simply about pioneering a new frontier - it is about sustaining excellence when the stakes are maximal.

## Future Directions: From Case Reports to Protocols

What is urgently needed in this field is more than experience - it is evidence. Current literature confirms what we intuitively suspect: smaller weight correlates with higher risk. But granular, stratified data on optimal timing, cannulation strategies, bypass modifications, and ICU protocols are sparse. Each center's anecdotal wisdom must be pooled, codified, and validated.

We are still in the case-report phase of micronatal cardiac surgery. With time and collaboration, we must evolve toward registries, prospective studies, and eventually consensus guidelines. In this endeavor, honesty is essential. Institutions must report both successes and failures transparently. Only then can the field mature responsibly.

## A Call to Purpose

This is a professional challenge. Are we as a community willing to confront the full demands of this delicate population? Will we invest in the infrastructure, training, and collaboration required to make survival after a Jatene procedure (arterial switch operation) in an 800-gram neonate not a miracle - but a metric? Micronatal cardiac surgery is not just a test of surgical dexterity - it is a test of institutional integrity. The smallest patients teach us the biggest lessons: about precision, preparation, and the sanctity of human life at its most fragile.

## Artificial Intelligence Usage

The authors declare use of Mendeley to organize references, Grammarly for grammar and syntax review, and ChatGPT to proofread the manuscript. No original content was produced by any artificial intelligence tool.
